# The Clicking Elateroidea from Chinese Mesozoic Deposits (Insecta, Coleoptera)

**DOI:** 10.3390/insects11120875

**Published:** 2020-12-09

**Authors:** Jyrki Muona, Huali Chang, Dong Ren

**Affiliations:** 1 Zoology Unit, Finnish Museum of Natural History, University of Helsinki, 00014 Helsinki, Finland; 2 Henan Geological Museum, Jinshuidonglu 18, Zhengdongxin Dsitrict, Zhengzhou 450016, China; changxinyin07@gmail.com; 3 College of Life Sciences and Academy for Multidisciplinary Studies, Capital Normal University, Xisanhuanbeilu 105, Haidian District, Beijing 100048, China; rendong@cnu.edu.cn

**Keywords:** Mesozoic, Elateroidea, fossils, synapomorphy, phylogeny, Eucnemidae, Throscidae, Elateridae

## Abstract

**Simple Summary:**

Fossil click beetles from the family Elateridae are frequent in Mesozoic deposits, especially in Russia and China. In order to understand their relationship to extant species, we need to search for evolutionary important characters that unite these dinosaur-era beetles with extant forms. We show that about one third of the identifiable click-beetles described from Mesozoic Chinese deposits are in fact members of other related families. Our results strengthen the view that these beetle groups are significantly older than often assumed, being present in the Early Jurassic. This is an important result as it indicates that molecular studies based on modern species do not replace paleontology—both are needed, even in the future.

**Abstract:**

Recent molecular studies have suggested that the clicking beetle families Elateridae, Eucnemidae, Throscidae, and Cerophytidae evolved in the Jurassic and diversified in the Cretaceous. These studies paid little attention to fossils, using them only as dating tools. The identification of Elateridae fossils is challenging, as external synapomorphies are not known for this family. Elateridae can be identified only as something not belonging to the other related families, all of which have diagnostic synapomorphies. Most subfamilies and tribes of Elateridae do possess definite diagnostic characters, however, making their identification feasible. We checked the 28 Elateridae described from Chinese Mesozoic deposits. Twelve were Elateridae, seven were Eucnemidae, and one was a Throscidae. Three species could be Eucnemidae, but showed aberrant characters. Five species could not be placed and may not belong to Elateroidea at all. On the basis of these results we suggest that all previously described Elateridae fossils should be re-checked. They should be searched for synapomorphies defining Eucnemidae, Throscidae, and Cerophytidae. If such characters are not present, a click beetle type of fossil can be placed in Elateroidae *incertae sedis*. The Mesozoic Chinese Elateridae fossils all belong to clades that do not exist today, whereas the Mesozoic Eucnemidae subfamilies are extant ones. This may be the source of the disagreement between Elateridae fossil age and datings based on molecular studies. One new combination was made: *Desmatus ponomarenkoi* (Chang, Kiretjshuk & Ren, 2009) NEW COMBINATION (= *Paradesmatus ponomarenkoi* Chang, Kirejtshuk & Ren, 2009).

## 1. Introduction

Elateroidea fossils with a promesosternal clicking mechanism are frequent in Cretaceous and Jurassic deposits [1,2,3,4]. Extant forms with this structure have been placed invariably in four families: Cerophytidae, Elateridae, Eucnemidae, and Throscidae [5]. We share the view that in the context of morphology and anatomy, only analytically recovered synapomorphies can be used for defining clades. The strength of this approach is that once a phylogenetic hypothesis has been formed, new taxa, extant or extinct, showing the relevant characters can be placed safely. In addition, all character combinations contradicting previously obtained hypotheses indicate the need for a new analysis and produce a new hypothesis.

Analytically obtained morphology-based phylogenetic hypotheses for clicking Elateroidea families exist for Eucnemidae [6,7], Throscidae [8], and Cerophytidae [9]. Douglas [10] provided the only morphology-based attempt to analytically study the monophyly of Elateridae. He did not recover any unique synapomorphies defining Elateridae, nor have such features been suggested in non-analytical studies. Most taxa traditionally classified as belonging to Elateridae have a bursa with colleterial glands (see especially [11]), a feature not present in other clicking Elateroidea. Muona & Teräväinen [12] suggested that this might turn out be an Elateridae synapomorphy. Naturally, when studying fossils this character is of no practical use.

The study of Elateroidea fossil material belonging to these four families has been hampered by the fact that there are no known external evolutionary novelties defining the most specious group, Elateridae. In paleontology this meant that Eucnemidae, Throscidae, and Cerophytidae could be identified from well-preserved samples, but Elateridae could be defined only as clicking elateroids not belonging to the three other families. This suggests that the monophyly of Elateridae should be critically scrutinized. Interestingly, five of the seven recent analyses based on molecular data failed to recover the Elateroidea families in the traditional sense [13,14,15,16,17].

Researchers have often relied on the “overall look” when classifying elateroid fossil taxa. This seemed to work with respect to Throscidae, as these beetles were characteristic in form and structure and possessed one visible synapomorphy, peculiar antennal grooves [8,18]. Members of the family Cerophytidae tended to be recognizable as well due to close antennal insertions and body form. The monophyly of Cerophytidae was questioned by both Chang et al. [19] and Oberprieler et al. [4] on the basis of fossil material, but Yu et al. [9] were able to demonstrate the presence of a unique prothoracic synapomorphy for cerophytids. The fourth clicking elateroid family, Eucnemidae, is closest to Elateridae in general appearance. Contrary to elaterids, the extant eucnemids can be defined by several synapomorphies, both external and internal, as well as in adults and larvae [6,20].

It is important to keep in mind that apomorphy, plesiomorphy, and homoplasy are data- and analysis-dependent relative factors. All plesiomorphies have once been apomorphies and many apomorphies are also homoplasies, depending on the taxa included in the sample. Any single character, taken out of context, i.e., the result of a specific analysis, can be misleading when used in another context, e.g., an analysis of a different data set. Searching for synapomorphies from fossils must be based on a previously established analytical hypothesis of the group in question.

## 2. Materials and Methods

The external characters for recognizing eucnemids from other clicking elateroids, especially elaterids in the context of the analytical hypotheses of Muona [6,8] and Lawrence et al. [7] are:

(1) Antennomere 2 attached subapically to antennomere 1 (i.e., pedicel to scape).

In Elateridae the attachment is usually apical, with Lissominae and Thylacosterninae, two highly characteristic groups with no described fossils being an exception. Throscidae have apically attached antennomere 2; Cerophytidae subapically attached antennomere 2.

In the text we shorten “antennomere” to “a”, e.g., “antennomere 1” is written “a1”.

**Figure 1:**
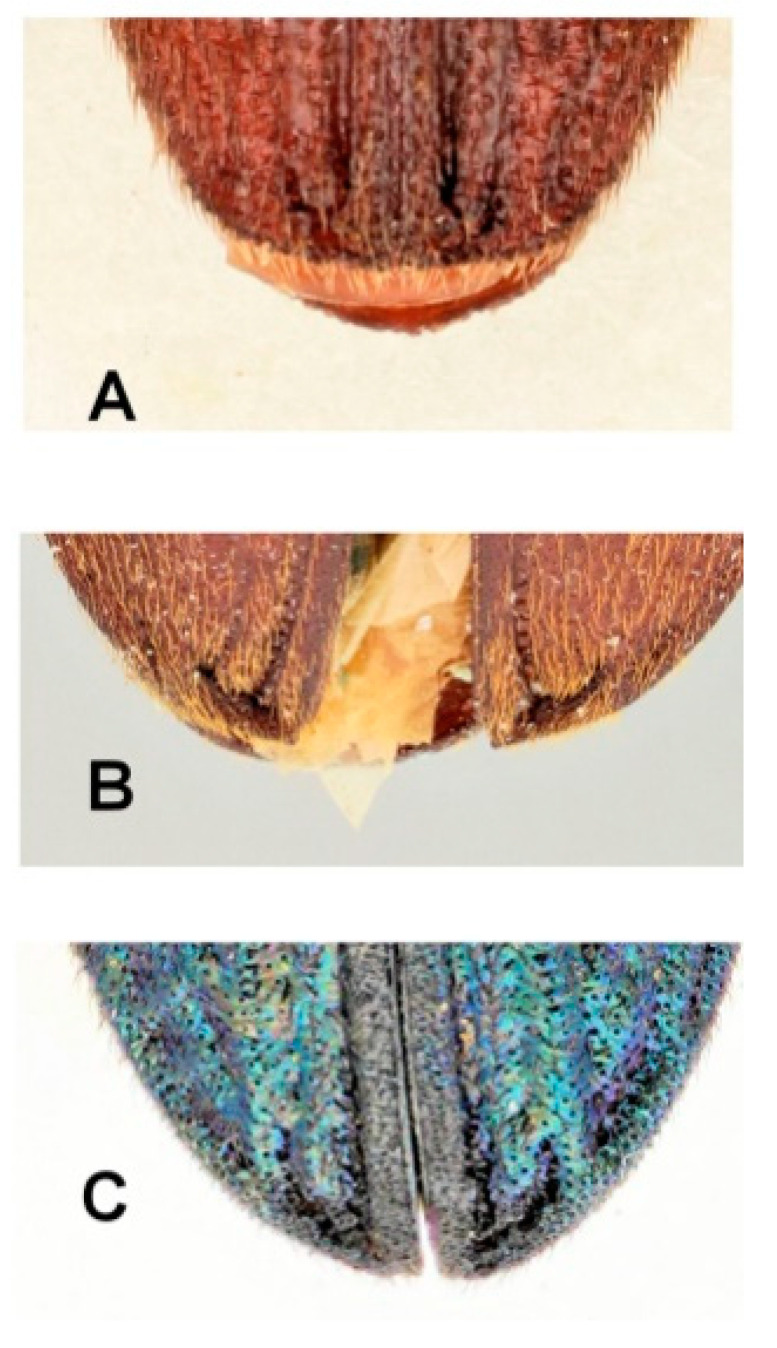
Eucnemidae. Elytral apices with specialized pores on the first and outmost striae apically. (**A**). *Euryptychus* sp. (**B**). *Phaenocerus subclavatus* Bonvouloir. (**C**). *Calyptocerus irdis* Otto.

(2) Elytral apices have specialized enlarged pores along the first interstices, often extending towards the front along the elytra laterally or present only there (Figure 1).

This feature has turned out to be an excellent synapomorphy for identifying all derived eucnemids [20]. It is present in Eucnemidae *sensu* Muona [6] except for the two most basal subfamilies Perothopinae and Phyllocerinae. It is a synapomorphy for all the lignicolous eucnemids, the clade “Eucnemidae from Pseudomeninae on” [12,20]. It is rarely reversed to an unmodified state in a few derived Macraulacini genera (e.g., *Euryaulacus* Bonvouloir, *Nodema* Fleutiaux, and some *Scython* Laporte species).

This feature is rarely found in Elateridae as well ([10], character 90). Although it has been known for a long time that Eucnemidae have special porous elytral structures, e.g., in the genus *Galbites* Fleutiaux [21], it was not before Otto & Gruber [22] discussed this feature with reference to the genus *Stethon* LeConte that the true significance of these pores became obvious. This was a great discovery as it provided a new external synapomorphy to recognize the lignicolous fossil Eucnemidae. The importance of their discovery cannot be overemphasized, as single elytron finds of fossil elateroids are common.

(3) Labrum attached underneath the expanded frontoclypeal region.

In most eucnemids the labrum is fully concealed and membranous. It is small, hard, and visible in *Anischia* Fleutiaux, partly sclerotized, dark and densely hairy in *Melasis* Fabricius and *Compsocnemis* Bonvouloir, hard, and partly visible in *Xylotho* Lucht and a few undescribed South American forms. It is well developed and partly visible in several undescribed Burmese amber species (JM, in prep.).

Cerophytidae have a concealed labrum as well.

Throscidae have an apically attached free, well-sclerotized labrum, similar to that of Elateridae, but often very small.

Morphologically the attachment point is the same in all these families—Apical margin of clypeus —but in Eucnemidae and Cerophyidae, the frontoclypeal region has expanded and turned ventrally forming a cavity enclosing the mouth region between it and the mandibles, pushing the true front margin and the labrum to the roof of the cavity, either entirely or partly.

(4) All visible abdominal ventrites connate.

The last visible one is freely movable in all Elateridae. This character is useful mainly when the state free ventrite can be actually seen; the fused state being difficult to observe from fossils. Eucnemidae: *Anischia* Fleutiaux has three apical ventrites free, Eucnemidae: Phyllocerinae has the last ventrite free, the rest of Eucnemidae and Throscidae have them all connate. Cerophytidae have several free abdominal ventrites.

(5) Lateral margins of hypomera with antennal grooves.

*Subprotelater* Fleutiaux is the only elaterid genus with grooves a bit similar to these, but they are shorter, extending about half-way to the hind angles of hypomera, a feature known only in one fossil eucnemid genus, the Eocene *Potergites* Britton. Compression fossils with this character are not known this far, but the feature can be observed in samples from Burmese and Baltic amber. Cerophytidae lack antennal grooves, and the unique synapomorphic Throscidae antennal grooves run close to the notosternal suture.

These antennal grooves characterize numerous genera in Eucnemidae subfamilies Macraulacinae and Eucneminae.

(6) Antennae with three enlarged apical antennomeres (i.e., flagellomeres 7 to 9).

Elateridae and Cerophytidae do not have enlarged apical antennomeres; Throscidae have an apical antennal club with three or four enlarged apical antennomeres.

This is a synapomorphy for lignicolous Eucnemidae, reversed in most extant lineages.

(7) One or two protibial apical spurs.

The basal eucnemids and most elaterids have two protibial spurs. All eucnemids belonging to subfamilies Macraulacinae, Eucneminae, and Melasinae have only one such spur. This character is difficult to see in compression fossils but easy to observe in Amber fossils. Both families include a few forms with no apical protibial spurs. Throscidae do not have visible tibial spurs.

(8) Male protarsomere one with sex-comb.

Not present in other Elateroidea. This character can be difficult to see from compression fossils, but it is a useful character for amber fossils.

This is a synapomorphy for Melasinae: Dirhagini as well as basal Macraulacinae and Eucneminae, lost in ancestor to all derived extant Eucneminae.

(9) Pronotal hind angles without short sublateral carina.

In Eucnemidae these carinae appear only in some small Dirhagini (e.g., *Microrhagus* Fabricius). In Elateridae they occur in the majority of the species. Cerophytidae and Throscidae do not have these structures. This is a good diagnostic feature separating most elaterids from most eucnemids. Species in all families may have a lateral carina separating the dorsal side of the pronotum from the ventral side, the hypomera.

(10) Mesothoracic sclerites fused.

The sclerites are free in *Perothops* Latreille, fused in most other Eucnemidae. Other Elateroidea have these sclerites free. This character is usually very hard to determine in Elateridae and Eucnemidae, as the edges of the mesanepisterna, mesepimera, and mesoventrite appear open in both cases. The fused suture between mesanepisternum and mesepimeron is usually easiest to observe. The free state in Throscidae is always obvious.

Oberprieler et al. [4] suggested a novel eucnemid character, stating: “However, the structure of its clicking apparatus (in new genus *Beattieellus*), featuring a broad and short mesoventral cavity flanked by a pair of prominent tubercles, is also characteristic of Eucnemidae. In eucnemids the lateral rims of this cavity are generally angled in about the middle, the anterior part sloping upwards, and the angle is often strengthened into a short flange, callus or tubercle (Figure 13, arrow), whereas in Elateridae the cavity is typically longer and narrower and its lateral rims are not angled or produced in the middle”.

We have not had the opportunity to study the distribution of this feature to the required extent but it could be very useful as this part of the venter is often visible in fossils.

When assessing the material, our approach is as follows. Species with promesothoracic clicking apparatus are placed in Eucnemidae or Throscidae if known synapomorphies for either family are present. For Eucnemidae characters 1, 2, or 3 in any combination of at least two are regarded as definite proof and 9 and 10 are possible signs. Characters 4 and 6 show that either Eucnemidae or Throscidae is in question. Characters 5, 7, and 8 are definite Eucnemidae features, but they are not known from any fossils older than Burmese amber, deposited 99 million years ago. The placement and structure of the antennal groove (character 5) is either a Throscidae or Eucnemidae synapomorphy, depending on its type.

If a clicking elaterid-like species cannot be placed in Eucnemidae, Throscidae or Cerophytidae, it should be placed in Elateroidea *incertae sedis*.

We have studied the type material of all Chinese Mesozoic Elateridae species placed in the collections of the Capital Normal University, Beijing. Additionally, we attempted to interpret the remaining Chinese fossil species by studying the original descriptions and inspected the description of one Australian fossil.

## 3. Results

### 3.1. Species Belonging to Eucnemidae

***Anoixis complanus*** Chang, Kirejtshuk & Ren, 2010

Jehol biota, Yixian, Lower Cretaceous, China. Holotype.

Several features supported placement in Eucnemidae: Labrum was not visible, elytral apices had specialized pores, pronotal hind angles had no carina, and a2 was attached subapically to a1. The mesosternal sclerites appeared to be fused as well. The wide, robust body, and relatively large body size (9.3 mm) suggested subfamily Palaeoxeninae. Apical antennomeres were missing, however, and as the area around prosternal process was deformed, the high median part of typical Palaeoxeninae prosternum could not be evaluated. *Anoixis* is placed in Palaeoxeninae, as it is the likely position for it amongst Eucnemidae.

***Apoclion clavatus*** Chang, Kirejtshuk & Ren, 2010

Jehol biota, Yixian, Lower Cretaceous, China. Type-species for *Apoclion* Chang, Kirejtshuk & Ren, 2010. Holotype.

Several features clearly supported placement in Eucnemidae: Labrum was not visible, elytral apices had extensive specialized pores, pronotal hind angles had no carina, and a2 was subapically attached to a1. Antennomeres 9–11 were gradually enlarged, forming a club.

The general habitus of this species and the listed characters suggested placement in the eucnemid subfamily Pseudomeninae sensu Muona [6]. On the basis of the adult beetle structure, this subfamily has two tribes, both defined by apomorphies, Pseudomenini (highly derived male aedeagus) and Schizophilini (trilobed clypeal edge). *Apoclion* was plesiomorphic with respect to these features but apomorphic as to the antennal structure. The enlarged apical antennomere was an important early Eucnemidae synapomorphy from Paleoxeninae on [6,20]. Its presence in *Apoclion* (and *Paradesmatus*) suggested a relationship with Palaeoxeninae as well. Muona & Teräväinen [20] showed that the larva of *Schizophilus* Bonvouloir shared two synapomorphies with the larva of *Palaeoxenus* Horn (fused labrum, laterally serrate mandibles). They split the subfamily Pseudomeninae in two, resulting in a new hypothesis: Pseudomeninae (Schizophilinae (Palaeoxeninae (Melasinae (Eucneminae, Macraulacinae)))) [20]. The description of *Apoclion clavatus* showed the simple trilobed aedeagus, a plesiomorphic feature found in Schizophilinae and Palaeoxeninae but different, apomorphic, in Pseudomeninae. Without larval characters it was not possible to know whether *Apoclion* belonged to Schizophilinae or Palaeoxeninae. Based on the elongate, small body as well as the presence of typical wide-bodied Palaeoxeninae in the same stratum, *Apoclion* is placed in Schizophilinae.

***Apoclion antennatus*** Chang, Kirejtshuk & Ren, 2010

Jehol biota, Yixian, Lower Cretaceous, China. Holotype.

**Figure 2:**
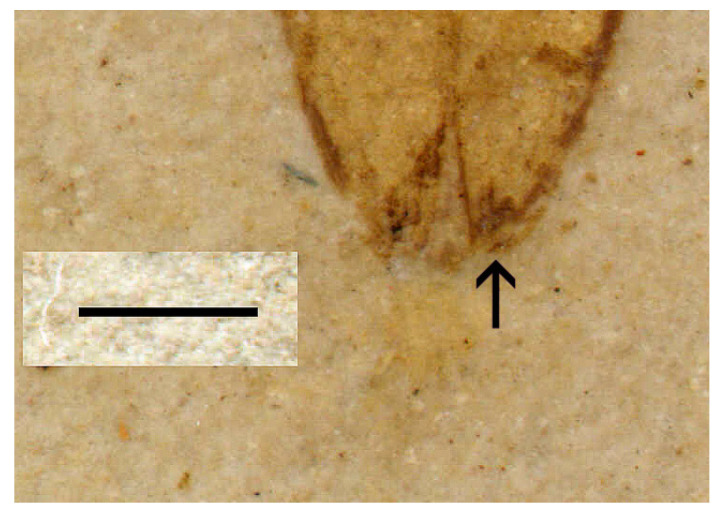
*Apoclion antennatus* Chang, Kirejtshuk & Ren, holotype. Elytral apices with specialized pores (arrow). Scale 1 mm.

**Figure 3:**
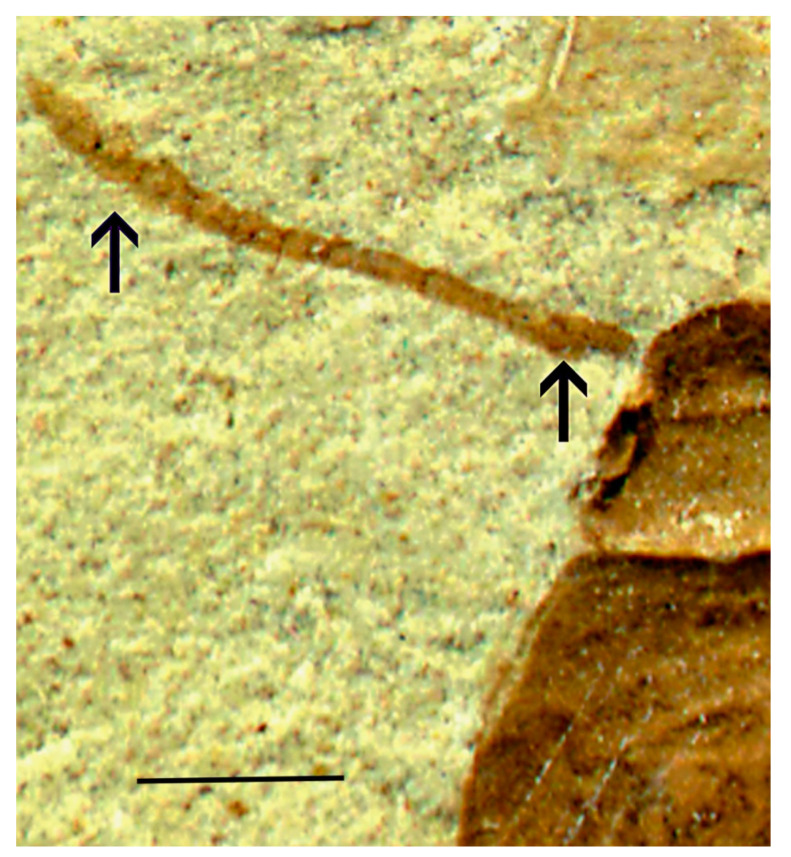
*Apoclion antennatus* Chang, Kirejtshuk & Ren, holotype. Antenna with subapically attached antennomere 2 (right arrow) and three enlarged apical antennomeres (left arrow). Scale 1 mm.

**Figure 4:**
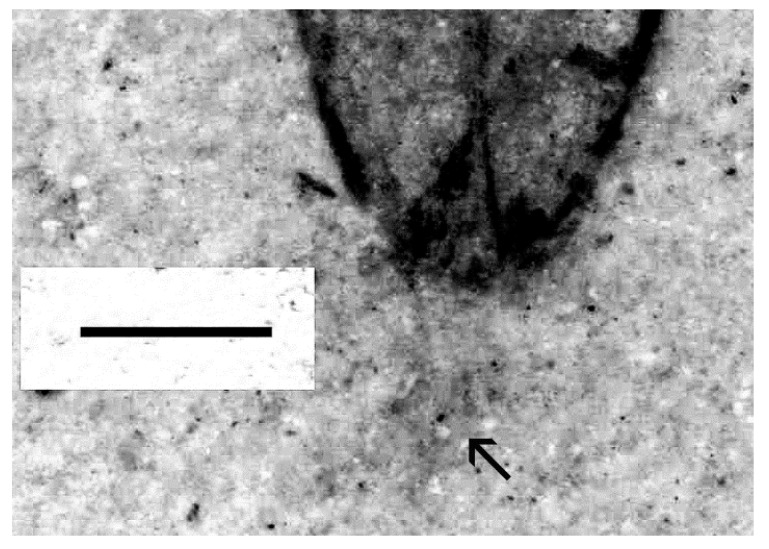
*Apoclion antennatus* Chang, Kirejtshuk & Ren, holotype. Elytral apex, simple trilobed aedeagus exposed (arrow). Scale 1 mm.

Several features clearly supported generic placement in Eucnemidae and Schizophilinae: Labrum was not visible, elytral apices had specialized pores (Figure 2), pronotal hind angles had no carina, a2 was attached subapically to a1, antennomeres 9–11 formed a club (Figure 3), and the body is small and elongated, similar to present-day *Schizophilus*. The male genitalia were of a simple trilobed type (Figure 4); originally placed in *Apoclion* and we see no reason to remove it from that genus.

***Apoclion dolini*** Chang, Kirejtshuk & Ren, 2010

Jehol biota, Yixian, Lower Cretaceous. Holotype.

**Figure 5:**
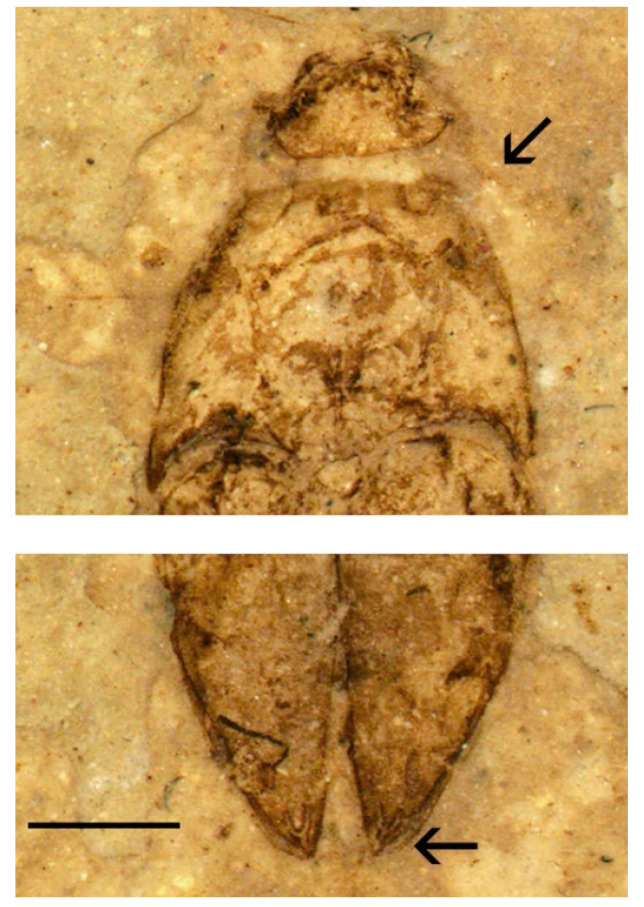
*Apoclion dolini* Chang, Kirejtshuk & Ren, holotype. Head and part of prothorax, antenna with subapically attached antennomere 2, and three enlarged apical antennomeres (upper arrow); apex of elytra with excretory punctures (lower arrow). Scale 1 mm.

Several features supported placement in Eucnemidae and Schizophilinae: Labrum was not visible, elytral apices had specialized pores (Figure 5), pronotal hind angles had no carinae, a2 was attached subapically to a1, apical antennomeres formed a club (Figure 5), and the body is small and relatively elongated. The remains of both antennae, including the club of the right one, were present in the holotype, but this was not mentioned in the text or present in the drawing in the original description ([3], Figure 4a); originally placed in *Apoclion* and we found no reason to change this.

***Palaeoxenus sinensis*** Chang, Muona & Teräväinen, 2016

Jehol biota, Yixian, Lower Cretaceous, China. Holotype.

The highly characteristic apomorphic larva placed this species in Eucnemidae: Paleoxeninae [23]. Other undescribed Palaeoxeninae species exist, both from Cretaceous and Jurassic Chinese deposits as well as Burmese amber samples.

***Paradesmatus baiae*** Chang, Kirejtshuk & Ren, 2009

Jiulongshan Formation, Middle Jurassic, China. Type-species of *Paradesmatus* Chang, Kirejtshuk & Ren, 2009. Holotype and one paratype.

Several features supported placement in Eucnemidae: Labrum was not visible, elytral apices had specialized pores, pronotal hind angles had no carinae, a2 was attached subapically to a1, and antennomere 9 was enlarged ([2], Figure 3). The elongate and small body suggested that this species belonged to Pseudomeninae or Schizophilinae rather than Palaeoxeninae. The two species placed in *Paradesmatus*, *P. baie,* and *P. dilatatus* are similar to each other, and there was no evidence for placing them in two genera. This being the case, *Paradesmatus* is placed in the subfamily Schizophilinae because *Paradesmatus dilatatus* Chang, Kirejtshuk & Ren has a plesiomorphic aedeagus. The general habitus reminds us of *Apoclion*.

***Paradesmatus dilatatus*** Chang, Kirejtshuk & Ren, 2010

Jehol biota, Yixian, Lower Cretaceous, China. Holotype.

Several features supported placement in Eucnemidae: labrum was not visible, elytral apices had specialized pores, pronotal hind angles had no carinae, and a2 was attached subapically to a1. As the simple trilobed aedeagus was visible, this species does not belong to Pseudomeninae. It is placed in Schizophilinae. Only antennomere 11 is enlarged, suggesting the genus was variable in this respect as is the subfamily.

### 3.2. Species Belonging to Throscidae

***Archaeolus funestus*** Lin, 1986

Xiwan Mine, Upper Middle Jurassic, China. Type material not seen, discussion is based on Dong et al. [24].

Dong et al. [24] published a revised description of this species placing it in Elateridae: Protagrypninae. Their image 2 showed the only visible throscid synapomorphy known: Antennal groove running close to the notosternal suture and then turning towards the hind corners of the prothorax above the protibial groove, antenna still in place. As described, the antennae were distinctly clubbed as well, another typical throscid feature. With the throscid key provided by Muona [8], *Anchaeolus* runs to *Pseudothroscus* Muona, having no tarsal grooves and simple occiput. It differs from *Pseudothroscus* in having very sharp antennal striae, similar to those of *Tyrannothroscus* Muona, and being elongated and parallel-sided. The prosternal process is quite different, abruptly narrowing at the front coxae, lancet-shaped. On the basis of the apomorphic prosternal process, we consider *Archaolus* as a distinct Throscidae genus, the sixth extinct one in addition to the four extant ones [8].

***Lithomerus wunda*** Martin, 2010

Mintaja, Early Jurassic, Western Australia. Holotype not seen.

Figure 4A,C in Martin [25] suggest that this species is a throscid rather than an elaterid. The high median portion of prosternum would fit both protagrypninae elaterids and throscids, but the small, wide body suggested the latter. Figure 4C showed a typical throscid clubbed antenna still on the left side in its usual position in the antennal groove, curving apically towards side, craniad to the groove for front tibia. We included this non-Chinese fossil here as an example of the wide distribution of throscids in the Jurassic. The transfer of this species to another family is complicated by the fact that *Lithomerus cockerelli* Dolin, the type-species of the genus, is a protagrypninae elaterid. *L. wunda* must be placed in another genus before this transfer can be made. This is best done after a detailed inspection of the holotype and in connection with a study of the undescribed Chinese Cretaceous and Jurassic throscids.

### 3.3. Species Retained in Elateridae

***Artinama qinghuanensis*** Lin, 1986

Shijaba, Hunan Province, Lower Jurassic, China. Type material not seen, discussion is based on Dong et al. [24].

The antennae appear to have a2 attached apically to a1, suggesting Elateridae. No evidence for another view can be observed and we do not remove *Artinama* from Elateridae.

***Bilineariselater foveatus*** Chang & Ren, 2008

Jehol biota, Yixian, Lower Cretaceous, China. Holotype.

No Eucnemidae or Throscidae synapomorphies could be observed and many Elateridae features were present in this species: free labrum, a2 apically attached to a1, antennae without apical club, elytral apices without special pores, and last ventrite free.

***Cryptocoelus buffoni*** Dolin & Nel, 2002

Jehol biota, Yixian, Lower Cretaceous, China. Holotype not seen, discussion based on Dolin et Nel [26].

No Eucnemidae or Throscidae synapomorphies were present in this species. It had many features placing it in Elateridae: labrum free, a2 attached apically to a1, and pronotal hind angles carinate.

Note that *Crytocoelus* [27,28,29] is an incorrect subsequent spelling.

***Cryptocoelus giganteus*** Chang, Ren & Shih, 2007

Jehol biota, Yixian, Lower Cretaceous, China. Holotype.

This species showed features placing it in Elateridae: labrum free, a2 attached apically to a1, last ventrite free, protibiae with two spurs and pronotal hind angles carinate, and lacking all Eucnemidae and Throscidae synapomorphies.

***Cryptocoelus major*** Dolin & Nel, 2002

Jehol biota, Yixian, Lower Cretaceous, China. Holotype not seen, discussion based on Dolin et Nel [26].

This species shows features placing it in Elateridae: labrum free, a2 attached apically to a1 and pronotal hind angles carinate, lacking all Eucnemidae and Throscidae synapomorphies.

***Curtelater wui*** Chang & Ren, 2008

Jehol biota, Yixian, Lower Cretaceous, China. Holotype.

Features found in Elateridae were visible: free labrum, a2 apically attached to a1, antennae without apical club, elytral apices without special pores, last ventrite free, and mesothoracic sclerites free. No Eucnemidae or Throscidae synapomorphies were present.

***Desmatinus cognatus*** Chang, Kirejtshuk & Ren, 2010

Jehol biota, Yixian, Lower Cretaceous, China. Holotype.

This species did not show features characterizing Eucnemidae, Cerophytidae or Throscidae. It should be retained in Elateridae. A2 was attached apically to a1, mesothoracic sclerites were free, no elytral special pores could be observed, and the right pronotal hind angle (ventral view) appeared to have a carina. The genus *Desmatinus* should remain in Elateridae: Protagrypninae: Desmatini.

***Desmatus ponomarenkoi*** (Chang, Kirejtshuk & Ren, 2009) NEW COMBINATION

Described as *Paradesmatus ponomarenkoi* Chang, Kirejtshuk & Ren, 2009.

Jiulongshan Formation, Middle Jurassic, China. Holotype and one paratype.

This species did not show features characterizing Eucnemidae, Cerophytidae or Throscidae. It should be retained in Elateridae. As *P. baiae*, the type-species of the genus *Paradesmatus* belongs to Eucnemidae, *P. ponomarenkoi* requires another generic name. *Paradesmatus* was described as being closely related to *Desmatus,* and both *P. ponomarenkoi* and *P. baie* were described as being very similar to each other. At the present stage of fossil Elateridae taxonomy, we find it best to place this species in the genus *Desmatus* Dolin, 1975, as *Desmatus ponomarenkoi* (Chang, Kirejtshuk & Ren) new combination.

***Paralithomerus exquisitius*** Chang, Zhang & Ren, 2008

Jehol biota, Yixian, Lower Cretaceous, China. Holotype.

This species has no synapomorphies suggesting Eucnemidae or Throscidae, but showed a suite of Elateridae features: free labrum, a2 attached subapically to a1, antennae without club, and free last ventrite.

***Paralithomerus parallelus*** Chang, Zhang & Ren, 2008

Jehol biota, Yixian, Lower Cretaceous, China. Holotype.

This species had simple, striate elytral apices, and carinate pronotal hind angles. Although antennae are not present and the free labrum is not explicitly visible, this species can be safely placed in Elateridae as it lacks all synapomorphies characterizing Eucnemidae or Throscidae.

***Paraprotagrypnus superbus*** Zhang, Zhao & Ren, 2009

Jiulongshan Formation, Middle Jurassic, China. Holotype and one paratype.

This is one of the many elateroids described in the Elateridae subfamily Protagrypninae Dolin, 1973. This subfamily is characterized by a conspicuous median plate-like structure on the proventrite, a structure sometimes referred to as a “sclerite”. It is clear that morphologically no such separate sclerite exists in polyphagan beetles. After having studied dozens of specimens of several species of this type, we have come to the conclusion that this plate is partly an artefact. When viewed from the ventral side, the central part of the prosternum (forming caudally the prosternal process) is abruptly lower than the rest of the ventrite. In a compression fossil this creates an illusion of a separate structure. This feature is prominent in one extant eucnemid as well, i.e., *Palaeoxenus dohrni* (Horn). The possibility that it also corresponds to the equally positioned structure in Throscidae should not be forgotten. The relationships between protagrypnines, palaeoxininae eucnemids, and throscids may be closer than previously noticed.

This species did not show features characterizing Eucnemidae, Cerophytidae or Throscidae. It should be retained in Elateridae. A2 was attached apically to a1, mesothoracic sclerites were free, and no elytral special pores could be observed. Pronotal hind angles seemed to lack carina, however.

We retain *Paraprotagrypnus superbus* in Elateridae: Protagrypninae.

***Protagrypnus robustus*** Chang, Kireijtshuk & Ren, 2009

Jiulongshan Formation, Middle Jurassic, China. Holotype and two paratypes.

This species did not show features characterizing Eucnemidae, Cerophytidae or Throscidae. It should be retained in Elateridae. A2 was attached apically to a1, supporting this placement, and antennae were loosely segmented as in most elaterids. Pronotal hind angles seemed to lack carinae, however. We retain *Protagrypnus robustus* in Elateridae: Protagrypninae.

### 3.4. Species Showing Affinities with Eucnemidae and Elateridae

***Clavelater ningchengensis*** Dong et Huang, 2011

Jiulongshan Formation, Middle Jurassic, China. Holotype not seen, discussion is based on Dong & Huang [30].

General body form and prosternal structure suggest protagrypninae elaterids, but plate 2.2 in [30] shows that a2 is subapically attached to a1. Labrum is drawn as being separate (Figure 2) but this is not visible in the photographs ([30], plate 2.1–2). This species may represent a formerly unknown eucnemid clade.

***Gripecolous enallus*** Lin, 1986

Xiwan Mine, Upper Middle Jurassic, China Type material not seen, discussion is based on Dong et al. [24].

According to Figure 3 in [24] this species may have enlarged excretory pores close to the apex of elytra. Without examining the holotype this is not certain, but we suspect this species belongs to Eucnemidae. Other relevant features cannot be seen from the image, however. The body form and prosternal structures fit protagrypninae elaterids as well.

The specimen is female, not male, as the Figures 3 and 5 in [24] show an ovipositor, not male genitalia.

***Lithomerus buyssoni*** Dolin & Nel, 2002

Jehol biota, Yixian, Lower Cretaceous, China. Holotype not seen, discussion based on Dolin & Nel [26].

The illustrations in the original description provide little information as such, so a renewed study of the holotype is in order. However, we point out the simple pronotal hind angles, a very unusual feature in Elateridae but common in Eucnemidae. Antennae are not visible; drawing 1 in the original description indicates the presence of a free labrum but the text states only: ”Tête presque quadrangulaire, partie antérieure du front largamente arrondie” [24]: 342.

This species could belong to Elateridae: Protagrypninae or Eucnemidae: Palaeoxeninae.

### 3.5. Species with Uncertain Status

The status of six species could not be assessed: *Fengningia punctata* Hong, 1984, *Mercata festira* Lin, 1986, *Microcoleus brunneus* Hong, 1984, *Ovivagina longa* Zhang, 1997, *Sinoelaterium melanocolor* Ping, 1928, and *Sinolithomerus dolini* Dong & Huang, 2009. A study of the type material is needed, and this was not possible because of logistic problems at this time.

A Tongchuan Elateriformia sample from Triassic China, a taxon not formally described as of yet, deserves to be mentioned [31]. It has been listed as belonging to Elateriformia. On the basis of a high-resolution image it could be an early elateroid. The fossil appears to have a prosternal intercoxal process and expanded metacoxal plates. Interestingly, the short antennae show a distinct antennal club with three or four apical antennomeres. The general body form—possibly deformed, however, suggests Dascillidae or Eulichadidae more than any elateroids, but the antennal club brings basal Eucnemidae to mind. Hopefully this 237 Ma fossil can be properly placed in future.

## 4. Discussion

Kundrata et al. [32] published a detailed compilation of all Elateridae fossil taxa recently. This work is a much needed source for the species included in the family, their nomenclature, and the literature dealing with them. The authors added a discussion of the position of many dubious forms as well. This discussion is certainly useful.

We have attempted to evaluate the described Chinese Cretaceous fossils in order to find out whether they belong to Elateridae as described. External synapomorphies defining Elateridae are not known, and “diagnostic characters” used in such a situation have little evolutionary meaning, as the term includes both apomorphies and plesiomorphies. Our conclusion is that a character assumed to be typical of an Elateridae species should be used only after the synapomorphies of Eucnemidae, Throscidae, and Cerophytidae have been screened for and observed not to be present. Features such as a clicking mechanism, body form, acute pronotal hind angles, metacoxal plates, and prosternal chin-piece all occur in other elateroids besides elaterids, some always, and others rarely. An elaterid in the traditional sense is a beetle that does not possess synapomorphies of one of the three other clicking elateroid families. This is the unfortunate situation—external apomorphies are not available for placing a taxon in Elateridae.

There are some more inclusive clades classified as belonging to Elateridae that can be identified with highly likely synapomorphies. These include forms such as the clicking elaterid “subfamilies” Lissominae, Thylacosterninae, Agrypninae, and Cardiophorinae as well as the non-clicking clades Omalisinae and Drilini.

As suggested by Kundrata et al. [33] on the basis of a well-sampled molecular analysis, all these forms may belong together with clades such as Lampyridae, Cantharidae, and Lycidae. In fact, all remaining Elateridae may be part of that assemblage. This does not render Elateridae polyphyletic, of course, but makes it a more extensive clade than previously thought. Unfortunately, Elateridae then also becomes impossible to define externally.

Although the result presented in Kundrata et al. [33] was not strongly supported by the data used [20], it gained support from several later molecular studies, even if in various configurations [13,14,15,16,17]. This suggested that although the clicking ability was a synapomorphy for Elateroidea, Elateridae have diversified greatly, and some groups have lost the clicking mechanism several times during their evolution. This has rarely taken place within Eucnemidae as well [7].

This development in the Elateridae lineage will most likely lead to a situation where families including both clicking and non-clicking forms are going to be established, depending on the logic used for making classifications.

The details of these splits remain obscure for now.

Twenty-seven Mesozoic Elateridae species and one Mesozoic Eucnemidae species have been described from China this far. With an evolutionary approach based on synapomorphies, we have been able to show that many of them were misplaced (Table A1). Seven species belong to Eucnemidae, five species belong to Elateridae subfamily Protagrypninae, and five are Elateridae *incertae sedis*. Three species have character combinations suggesting both Eucnemidae and Elateridae. They may be eucnemids that belong to extinct lineages, but only a new global analysis can solve their placement. One species belongs to Throscidae and appears to be related to the extinct genus *Pseudothroscus* Muona. The position of five species remains unclear. Their status can be solved only by studying the types.

These results changed drastically our view of the Mesozoic elateroid fauna of China. Cerophytidae had been shown to exist in Jurassic Chinese deposits earlier [19]. We show that Eucnemidae, Throscidae, and definitely Elateridae in the traditional plesiomorphic sense existed in the Jurassic as well. An important point to note is that none of the Mesozoic Chinese Elateridae fossils belong to extant elaterid groups, whereas most if not all the Eucnemidae can be placed in basal extant subfamilies, Schizophilinae and Palaeoxeninae. These facts highlight the problems in trying to elucidate Elateridae phylogeny on the basis of extant forms only.

Chang et al. [3], when describing Desmatini elaterids, suggested that they might belong to Eucnemidae. This idea was taken up, surprisingly as if new in Li et al. [34]. They suggested a novel separating feature for Eucnemidae, the form of the metacoxal plates, “a critical eucnemid character”. This feature varies within both genera and tribes of Eucnemidae and Elateridae and has not been quantified in an objective fashion. Another character they pointed out as a eucnemid feature was enlarged apical antennomeres. They had been shown to be of importance within Eucnemidae earlier, of course [6]. Within Elateroidea, the antennal club is useful only when taking into account the Throscidae as well. Li et al. [34] also suggested, on the basis of McKenna et al. [17], that it is more likely that Yixian formation elateroids are Eucnemidae rather than Elateridae. The existing literature of known fossils demonstrates that this claim has no basis [4,16,32,35]. The estimated ages of Elateroidea clades in McKenna et. al. [17] are off by tens of millions of years and with respect to Cerophytidae, Throscidae, and Eucnemidae give an incorrect picture of the evolutionary age of these clades as well as the age of Elateridae in a traditional sense.

## 5. Conclusions

Our re-evaluation of Cretaceous and Jurassic Elateridae fossils from China confirmed that the presence of Eucnemidae and Throscidae beetles had been overlooked when describing the material (see also [3]). It appears clear that the status of all fossil Elateridae species should be re-evaluated. All future work with Mesozoic elateroids should start from checking for external synapomorphies of other clicking Elateroidea families.

The Jurassic and Cretaceous presence of species belonging to eucnemid subfamilies Schizophilinae and Palaeoxeninae is of considerable interest. It offers further proof that the complex co-evolution of the eucnemid larvae, forest trees, and fungi developed long before flowering trees existed. The discovery of the *Palaeoxenus* larva from Yixian was an unexpected piece of evidence for this, proving that the special larval adaptations existed in the Cretaceous [23]. The present study extends this scenario to the Jurassic (see also [20]).

The taxa discussed in the present paper include Eucnemidae and Elateridae from the Middle Jurassic and Early Cretaceous and Throscidae from the Middle Jurassic and very likely the Early Jurassic as well. Earlier Cerophytidae have been shown to be common both in Lower and Upper Jurassic deposits [19].

An analytically formed hypothesis of Elateridae phylogeny including fossil material and demonstrating external morphological synapomorphies is urgently needed. If one can be produced, it will also help to clarify the placement of the non-clicking derived elateroids. When a more extensive set of species has been studied with respect to the critical characters, a combined analysis based on these new data sets can be done. This requires a new study of the large Russian fossil collections and formal descriptions of the many already known but still undescribed Chinese fossil forms.

## Appendix A

**Table A1:** List of Chinese Mesozoic clicking Elateroidea.

**Throscidae**
*Archaeolus funestus* Lin
**Eucnemidae**
Schizophilinae
*Apoclion clavatus* Chang, Kiretjshuk & Ren
*Apoclion antennatus* Chang, Kiretjshuk & Ren
*Apoclion dolini* Chang, Kiretjshuk & Ren
*Paradesmatus baiae* Chang, Kiretjshuk & Ren
*Paradesmatus dilatatus* Chang, Kiretjshuk & Ren
Palaeoxeninae
*Anoixis complanus* Chang, Kiretjshuk & Ren
*Palaeoxenus sinensis* Chang, Muona & Teräväinen
**Eucnemidae/Elateridae**
*Clavelater ningchengensis* Dong, Huang
*Gripecolous enallus* Lin
*Lithomerus buyssoni* Dolin, Nel
**Elateridae**
Protagrypninae
Protagrypnini
*Paraprotagrypnus superbus* Zhang, Zhao & Ren
*Protagrypnus robustus* Chang, Kirejtshuk & Ren
Desmatini
*Desmatinus cognatus* Chang, Kirejtshuk & Ren
*Desmatus ponomarenkoi* (Chang, Kirejtshuk & Ren) NEW COMBINATION
(=*Paradesmatus ponomarenkoi* Chang, Kirejtshuk & Ren, 2009)
Elateridae *incertae sedis*
*Artinama qinghuanensis* Lin
*Bilineariselater foveatus* Chang, Ren
*Cryptocoelus buffoni* Dolin, Nel
*Cryptocoelus giganteus* Chang, Ren & Shih
*Cryptocoelus major* Dolin, Nel
*Curtelater wui* Chang, Ren
*Paralithomerus exquisitius* Chang, Zhang & Ren
*Paralithomerus parallelus* Chang, Zhang & Ren
**Unknown placement**
*Fengningia punctata* Hong
*Microcoleus brunneus* Hong
*Ovivagina longa* Zhang
*Sinoelaterium melanocolor* Ping
*Sinolithomerus dolini* Dong, Huang
